# “The COVID-19 pandemic in BRICS: Milestones, interventions, and molecular epidemiology”

**DOI:** 10.1371/journal.pgph.0003023

**Published:** 2024-12-20

**Authors:** Stephanie van Wyk, Monika Moir, Anindita Banerjee, Georgii A. Bazykin, Nidhan K. Biswas, Nikita Sitharam, Saumitra Das, Wentai Ma, Arindam Maitra, Anup Mazumder, Wasim Abdool Karim, Alessandra Pavan Lamarca, Mingkun Li, Elena Nabieva, Houriiyah Tegally, James Emmanuel San, Ana Tereza R. Vasconcelos, Joicymara S. Xavier, Eduan Wilkinson, Tulio de Oliveira

**Affiliations:** 1 Centre for Epidemic Response and Innovation (CERI), School of Data Science and Computational Thinking, Stellenbosch University, Stellenbosch, South Africa; 2 BRICS-National Institute of Biomedical Genomics, Kalyani, West Bengal, India; 3 A.A. Kharkevich Institute for Information Transmission Problems of the Russian Academy of Sciences, Moscow, Russia; 4 Indian Institute of Science, Bengaluru, Karnataka, India; 5 Beijing Institute of Genomics, CAS Key Laboratory of Genomic and Precision Medicine, Chinese Academy of Sciences / China National Centre for Bioinformation, Beijing, China; 6 University of Chinese Academy of Sciences, Beijing, China; 7 Laboratório de Bioinformática, Laboratório Nacional de Computação Científica, Petrópolis, Brazil; 8 Princeton University, Princeton, New Jersey, United States of America; 9 KwaZulu-Natal Research Innovation and Sequencing Platform (KRISP), Nelson R Mandela School of Medicine, University of KwaZulu-Natal, Durban, South Africa; 10 Institute of Agricultural Sciences, Universidade Federal dos Vales do Jequitinhonha e Mucuri, Unaí, Brasil; 11 Instituto René Rachou, Fundação Oswaldo Cruz, Belo Horizonte, Brazil; GU: Georgetown University, UNITED STATES OF AMERICA

## Abstract

Brazil, Russia, India, China, and South Africa (BRICS) are a group of developing countries with shared economic, healthcare, and scientific interests. These countries navigate multiple syndemics, and the COVID-19 pandemic placed severe strain on already burdened BRICS’ healthcare systems, hampering effective pandemic interventions. Genomic surveillance and molecular epidemiology remain indispensable tools for facilitating informed pandemic intervention. To evaluate the combined manner in which the pandemic unfolded in BRICS countries, we reviewed the BRICS pandemic epidemiological and genomic milestones, which included the first reported cases and deaths, and pharmaceutical and non-pharmaceutical interventions implemented in these countries. To assess the development of genomic surveillance capacity and efficiency over the pandemic, we analyzed the turnaround time from sample collection to data availability and the technologies used for genomic analysis. This data provided information on the laboratory capacities that enable the detection of emerging SARS-CoV-2 variants and highlight their potential for monitoring other pathogens in ongoing public health efforts. Our analyses indicated that BRICS suffered >105.6M COVID-19 infections, resulting in >1.7M deaths. BRICS countries detected intricate genetic combinations of SARS-CoV-2 variants that fueled country-specific pandemic waves. BRICS’ genomic surveillance programs enabled the identification and characterization of the majority of globally circulating Variants of Concern (VOCs) and their descending lineages. Pandemic intervention strategies first implemented by BRICS countries included non-pharmaceutical interventions during the onset of the pandemic, such as nationwide lockdowns, quarantine procedures, the establishment of fever clinics, and mask mandates- which were emulated internationally. Vaccination rollout strategies complemented this, some representing the first of their kind. Improvements in BRICS sequencing and data generation turnaround time facilitated quicker detection of circulating and emerging variants, supported by investments in sequencing and bioinformatic infrastructure. Intra-BRICS cooperation contributed to the ongoing intervention in COVID-19 and other pandemics, enhancing collective capabilities in addressing these health challenges. The data generated continues to inform BRICS-centric pandemic intervention strategies and influences global health matters. The increased laboratory and bioinformatic capacity post-COVID-19 will support the detection of emerging pathogens.

## Background

The SARS-CoV-2 virus emerged more than four years ago, causing a then-novel type of pneumonia, now known as COVID-19, which was until May 2023 regarded as a public health emergency of international concern. Globally, >704M people were infected by the SARS-CoV-2 virus, causing >7M fatalities (as of July 2024) [1]. High counts in cases and fatalities were often reported by the world’s most populated and economically strained countries [2,3]. BRICS, represented by Brazil, Russia, India, China, and South Africa, constitute one of the most populous groupings in the world [3]. Brazil, India, and South Africa are among the countries that witnessed a high number of cases during the COVID-19 pandemic [3]. India and Brazil rank second and fifth in reported case numbers and fatalities, while Russia and South Africa rank tenth and 38th [1], respectively.

Compared to more economically advanced countries, BRICS face unique challenges that hamper their pandemic interventions [3,4]. Typically, BRICS navigate multiple viral syndemics. South Africa and Russia suffer ongoing Human Immunodeficiency Virus (HIV) epidemics [5–7], while Brazil faces ongoing Arboviral outbreaks [8], such as Zika [9], Chikungunya [10], Yellow Fever [11], and Dengue [12]. For China, the COVID-19 outbreak represented the third life-threatening viral outbreak within two decades and occurred after two major Avian influenza outbreaks, the H5N1 in 1997 and the H7N9 in 2013 [13,14]. The influence of these syndemics puts a severe strain on already burdened BRICS healthcare systems [15].

The BRICS’ Network for Genome Surveillance (NGS-BRICS) was established in 2021 to respond to ongoing viral and other syndemics. The NGS-BRICS generates and investigates molecular data of viral and other pathogenic organisms of public health importance. However, pre-existing socioeconomic concerns, such as civil and political unrest, limited access to adequate health care services [16–19], growing concerns about large-scale malnutrition [4], and the consequences of global warming [20,21], remain barriers to effective BRICS pandemic interventions.

BRICS represents a unique socioeconomic and demographic grouping, which accounts for ~42% of the world’s population, ~27% of the world’s land surface area, and ~23% of the world’s Gross Domestic Product (GDP). Given the importance of BRICS nations in the global health landscape, the scientific contributions, and seminal disease prevention measures put into effect during the COVID-19 pandemic, we set out to discuss pandemic intervention strategies implemented by BRICS nations for the first 33 months of the pandemic (1 December 2019 to 31 October 2022), representing the height of the COVID-19 pandemic. This information was complemented with genomic and epidemiological data to provide further insights into the BRICS pandemic progression. To better understand the genomic surveillance efficiency in these countries, we investigated the turnaround time from sample collection to data availability and the platforms and technologies used for genomic surveillance. These results provided insights into the available laboratory capacity that may support the detection of emerging SARS-CoV-2 variants, which can be employed for other pathogens surveillance of ongoing public health threats in BRICS.

## Methodology

### Pandemic timelines

Data for this study were retrieved from Our World In Data (OWID) [1] and the narrative-based literature studies (see in-text citing materials and associated references). These studies included peer-reviewed published articles, governmental and regulatory authorities’ websites and data repositories, and national and international news outlets published between 1 December 2019 and 31 October 2022. Data from website sources cited were preserved as screenshots and are available from 10.6084/m9.figshare.22778249. The timeline was illustrated using Power BI Timeline storyteller [22].

### Epidemiological analyses

We investigated epidemiological parameters for the BRICS countries between 1 January 2020 and 31 October 2022. For the Chinese results, only data from mainland China were considered for these investigations. The data on the estimated daily confirmed cases and the total count of deaths due to COVID-19 disease were accessed from the COVID-19 Data Repository by the Centre for Systems Science and Engineering at Johns Hopkins University [23] and retrieved through OWID [1] https://ourworldindata.org/). For comparative purposes, OWID data on the pandemic statistics for BRICS and the other economic groupings listed as high-, low-, low-middle-, and upper-middle-income countries were accessed on 31 October 2022 (**S1 Table**). The statistics on case numbers, deaths, and count of genome sequences submitted to GISAID included the following regions based on WHO-recommended region groupings: Latin America and the Caribbean, European and Central Asia, South Asia, East Asia and Pacific, and Sub-Sahara Africa (see text for details).

### Genomic investigation

We investigated SARS-CoV-2 genomic data generated by the BRICS countries from 1 January 2020 to 31 October 2022. Metadata, genome sequencing technologies, lineage identification, and time from sampling to GISAID submission were retrieved from the Global Initiative on Sharing Avian Influenza Data (GISAID; https://www.gisaid.org/) [24]. Time from sample reception to publication to GISAID was used to determine the turnaround time. For GISAID submission acknowledgements, please see the following Episet identifiers generated using GISAD doi.org/10.55876/gis8.221130wr (Brazil), doi.org/10.55876/gis8.221130md (Russia), 10.55876/gis8.221204hf (India), 10.55876/gis8.221204xh (China), doi.org/10.55876/gis8.221129bx (South Africa). For the Chinese results, data from mainland China were considered for these investigations.

We evaluated epidemic waves by grouping the circulating SARS-CoV-2 lineages into the following VOCs: the Alpha (B.1.1.7), the Beta (B.1.351), the Delta (B.1.617), the Gamma (P.1), the Omicron BA.1 (BA; 21K), Omicron BA.2 (21L), Omicron BA.3 (21M), Omicron BA.4 (22A), and Omicron BA.5 (22B). Figures were generated using R Studio.

### Ethics statement

The use of South African samples for sequencing and genome surveillance was approved by the University of KwaZulu-Natal Biomedical Research Ethics Committee (ref. BREC/00001510/2020); the University of the Witwatersrand Human Research Ethics Committee (HREC) (ref. M180832); Stellenbosch University HREC (ref. N20/04/008_COVID-19); the University of the Free State Research Ethics Committee (ref. UFS-HSD2020/1860/2710) and the University of Cape Town HREC (ref. 383/2020).

## Results

### A. Progression of the COVID-19 pandemic in BRICS: Pandemic intervention and milestones

**Fig 1:**
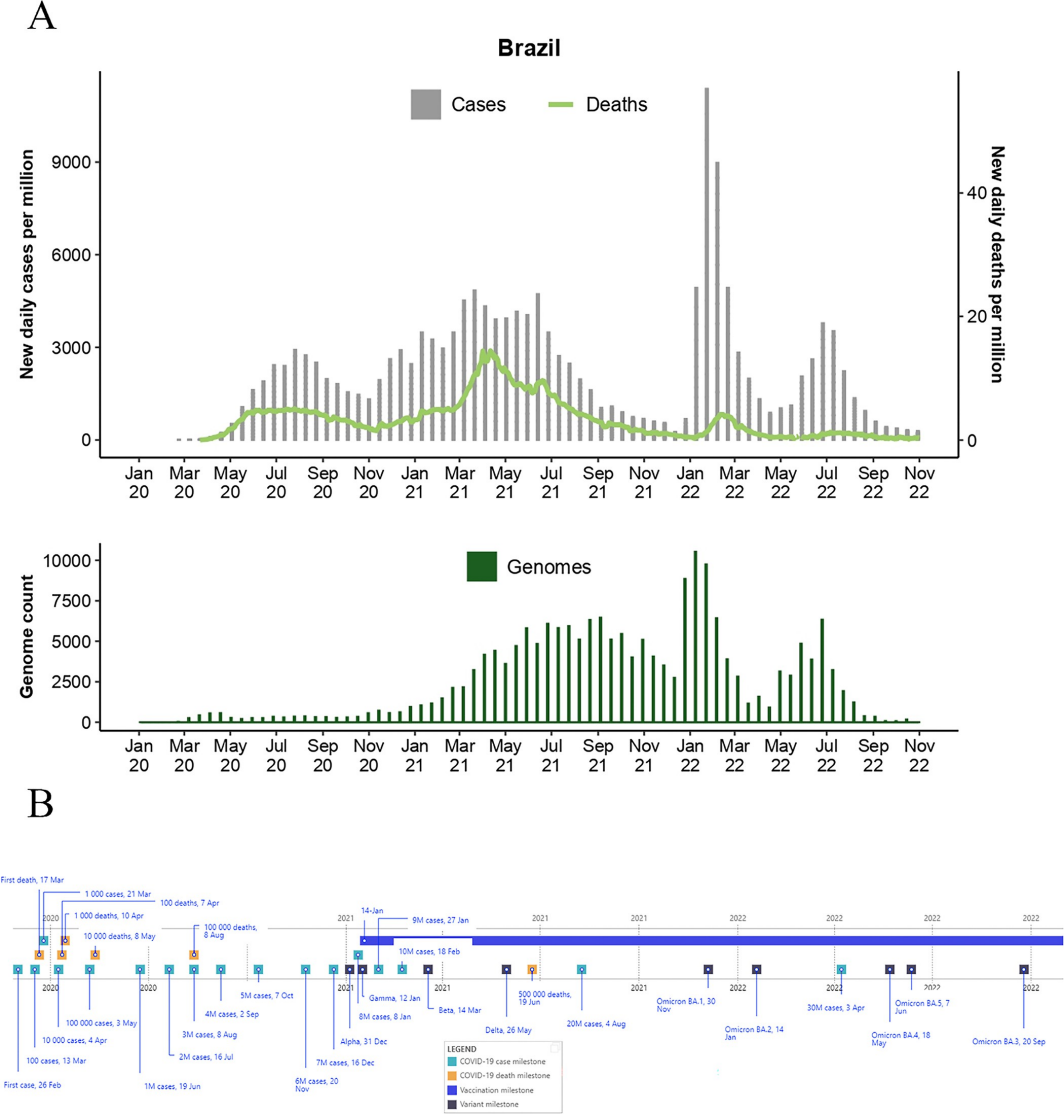
Epidemiological curve (top) and total genome assemblies (bottom) generated and deposited on GISAID by BRICS country Brazil. The graphs illustrate the daily number of COVID-19 cases (bars) and associated deaths (line graph) for BRICS countries from January 2020 to 31 October 2022 (accessed from the COVID-19 Data Repository by the Centre for Systems Science and Engineering at John Hopkins University and retrieved through Our World In Data (OWID; https://ourworldindata.org/) and genomic data were retrieved from Global Initiative on Sharing Avian Influenza Data (GISAID; https://www.gisaid.org/). **B)** Pandemic timeline illustration of the milestones for BRICS countries during the pandemic timeline (1 January 2020 to 31 October 2022) (accessed from the COVID-19 Data Repository by the Centre for Systems Science and Engineering at John Hopkins University and retrieved through OWID.

**Fig 2:**
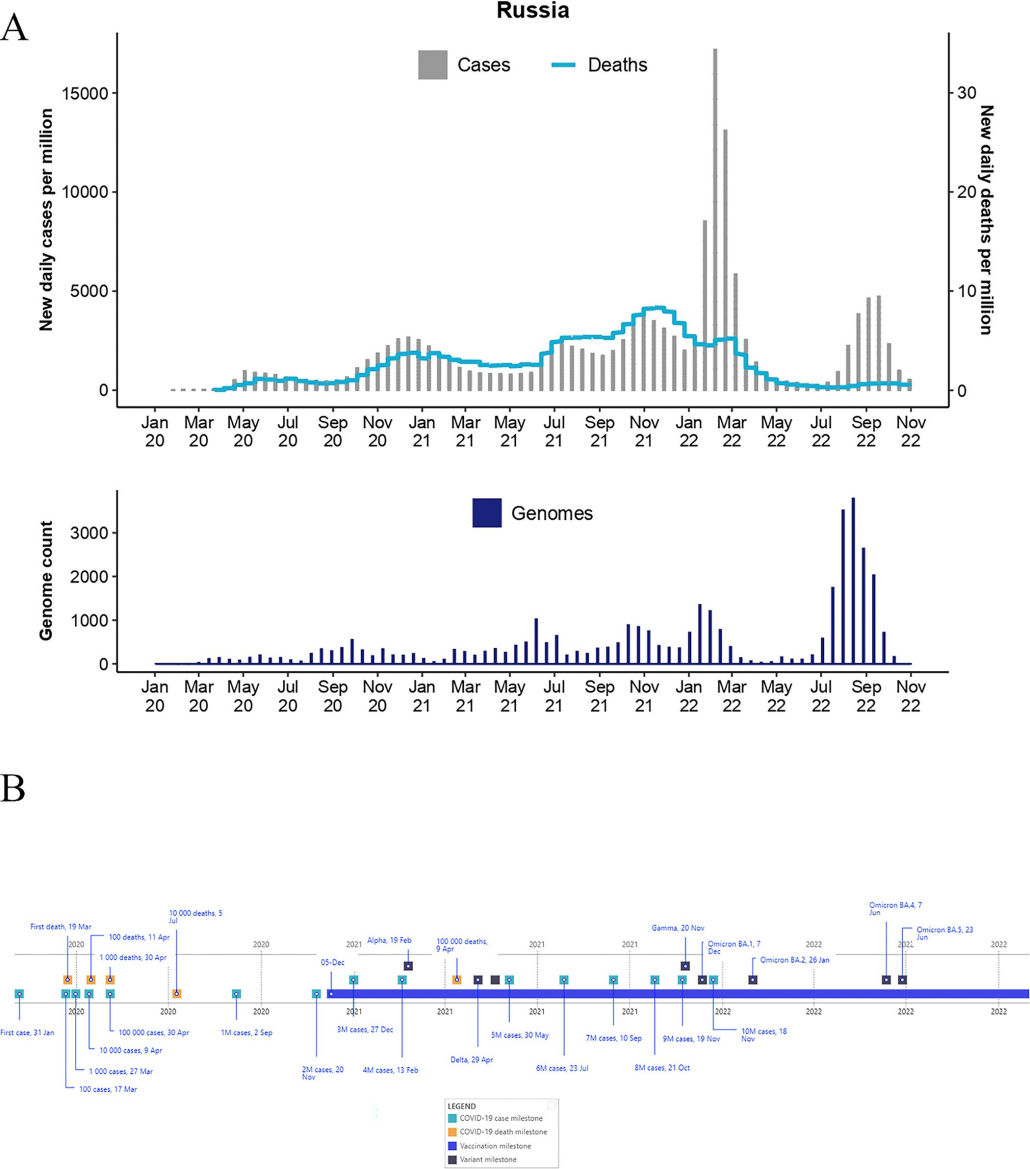
Epidemiological curve (top) and total genome assemblies (bottom) generated and deposited on GISAID by BRICS country Russia. The graphs illustrate the daily number of COVID-19 cases (bars) and associated deaths (line graph) for BRICS countries from January 2020 to 31 October 2022 (accessed from the COVID-19 Data Repository by the Centre for Systems Science and Engineering at John Hopkins University and retrieved through Our World In Data (OWID; https://ourworldindata.org/) and genomic data were retrieved from Global Initiative on Sharing Avian Influenza Data (GISAID; https://www.gisaid.org/). **B)** Pandemic timeline illustration of the milestones for BRICS countries during the pandemic timeline (1 January 2020 to 31 October 2022) (accessed from the COVID-19 Data Repository by the Centre for Systems Science and Engineering at John Hopkins University and retrieved through OWID.

**Fig 3:**
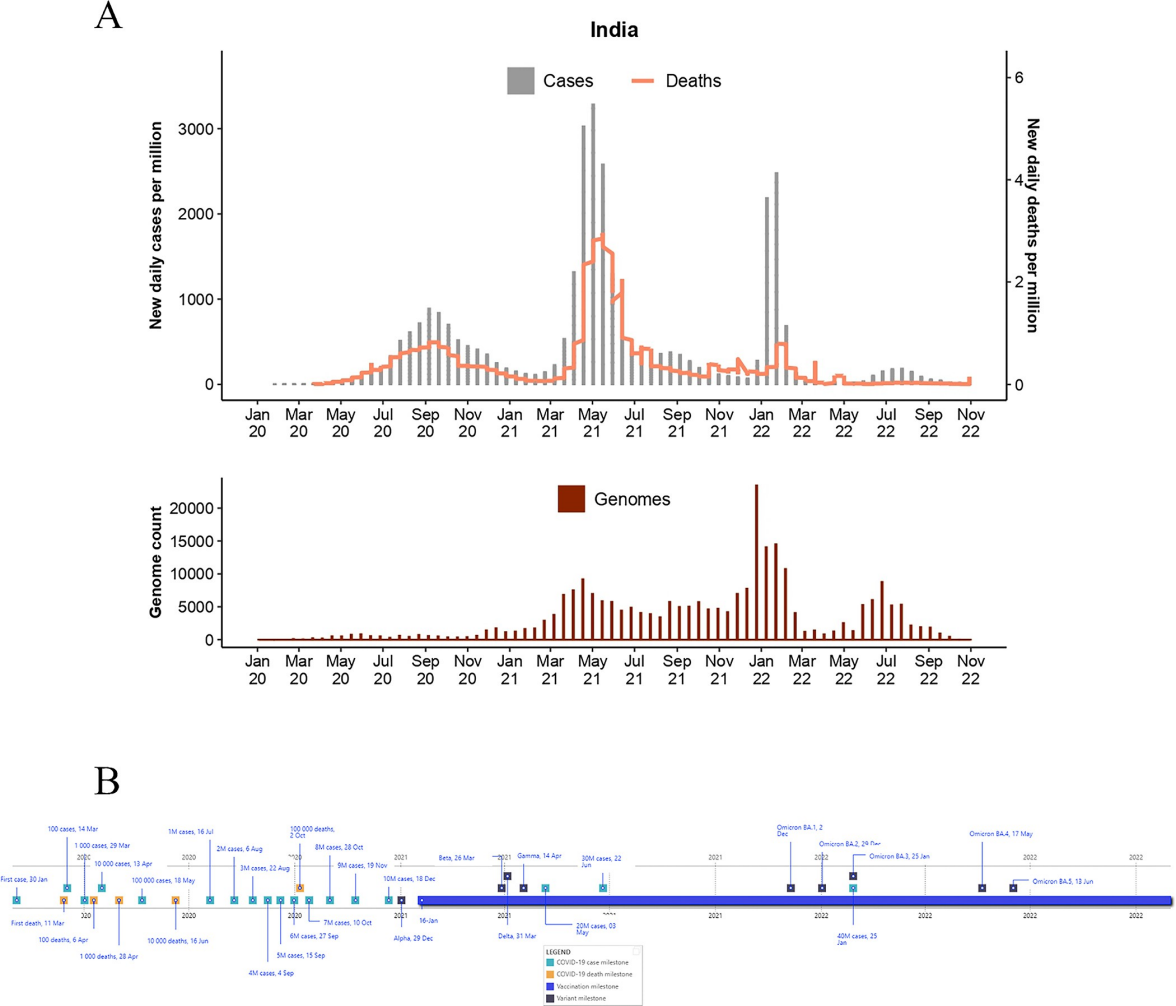
Epidemiological curve (top) and total genome assemblies (bottom) generated and deposited on GISAID by BRICS country India. The graphs illustrate the daily number of COVID-19 cases (bars) and associated deaths (line graph) for BRICS countries from January 2020 to 31 October 2022 (accessed from the COVID-19 Data Repository by the Centre for Systems Science and Engineering at John Hopkins University and retrieved through Our World In Data (OWID; https://ourworldindata.org/) and genomic data were retrieved from Global Initiative on Sharing Avian Influenza Data (GISAID; https://www.gisaid.org/). **B)** Pandemic timeline illustration of the milestones for BRICS countries during the pandemic timeline (1 January 2020 to 31 October 2022) (accessed from the COVID-19 Data Repository by the Centre for Systems Science and Engineering at John Hopkins University and retrieved through OWID.

**Fig 4:**
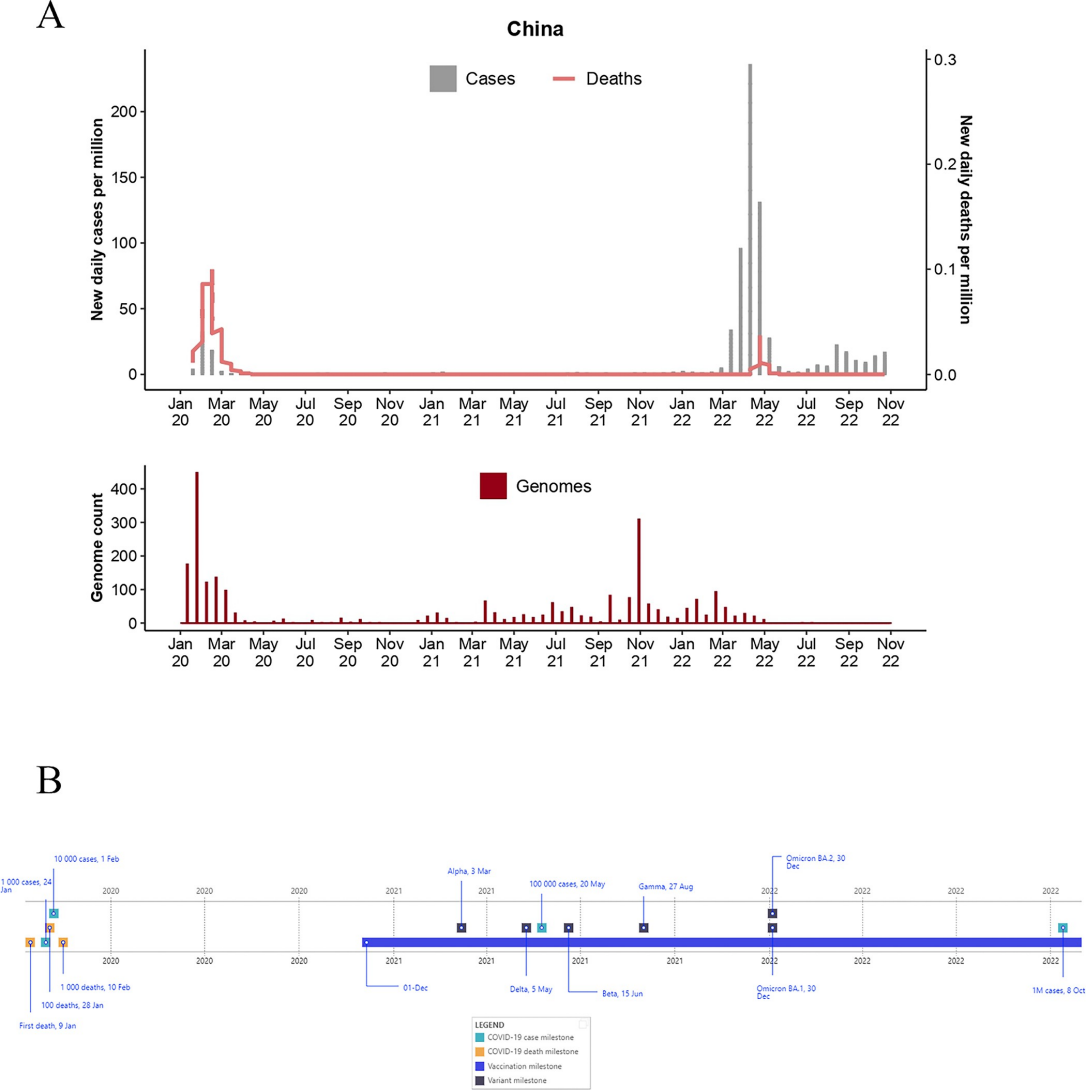
Epidemiological curve (top) and total genome assemblies (bottom) generated and deposited on GISAID by BRICS country China. The graphs illustrate the daily number of COVID-19 cases (bars) and associated deaths (line graph) for BRICS countries from January 2020 to 31 October 2022 (accessed from the COVID-19 Data Repository by the Centre for Systems Science and Engineering at John Hopkins University and retrieved through Our World In Data (OWID; https://ourworldindata.org/) and genomic data were retrieved from Global Initiative on Sharing Avian Influenza Data (GISAID; https://www.gisaid.org/). **B)** Pandemic timeline illustration of the milestones for BRICS countries during the pandemic timeline (1 January 2020 to 31 October 2022) (accessed from the COVID-19 Data Repository by the Centre for Systems Science and Engineering at John Hopkins University and retrieved through OWID.

**Fig 5:**
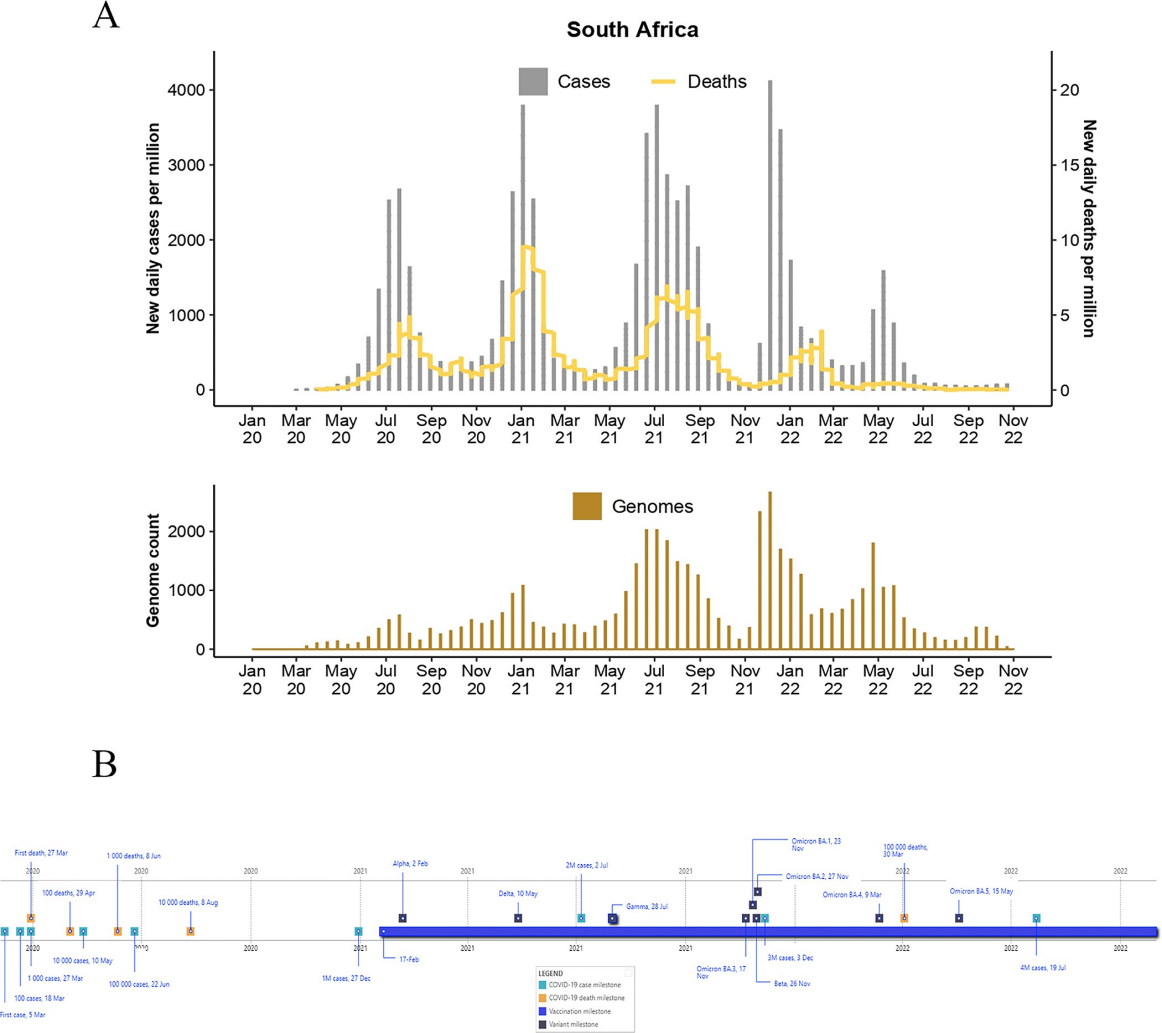
Epidemiological curve (top) and total genome assemblies (bottom) generated and deposited on GISAID by BRICS country South Africa. The graphs illustrate the daily number of COVID-19 cases (bars) and associated deaths (line graph) for BRICS countries from January 2020 to 31 October 2022 (accessed from the COVID-19 Data Repository by the Centre for Systems Science and Engineering at John Hopkins University and retrieved through Our World In Data (OWID; https://ourworldindata.org/) and genomic data were retrieved from Global Initiative on Sharing Avian Influenza Data (GISAID; https://www.gisaid.org/). **B)** Pandemic timeline illustration of the milestones for BRICS countries during the pandemic timeline (1 January 2020 to 31 October 2022) (accessed from the COVID-19 Data Repository by the Centre for Systems Science and Engineering at John Hopkins University and retrieved through OWID.

Overview:

The pandemic timelines (**Figs 1–5**) illustrate the progression of the COVID-19 pandemic for the first 33 months in the five BRICS nations, representing the height of the COVID-19 pandemic. Cumulatively, up to 31 October 2022, BRICS nations recorded >105.5 M cases and >1.7 M deaths [1] (**Tables 1 and 2**). Brazil and Russia recorded some of the world’s highest excess mortality rates throughout the pandemic, suggesting the recorded deaths may be greatly underestimated [25,26]. The COVID-19 cases in Brazil represented ~53.5% of cases recorded for the entire South American continent, India ~89% of cases from the South Asian regions, and South Africa ~32% of cases for the entire African continent [1]. In contrast, Russia represented only ~9% of cases of the total count from European countries, and China (mainland China), 1.9% of cases recorded in the East Asian regions.

**Table 1:** Comparative summary of BRICS statistics relating to the COVID-19 pandemic. The data below includes the total number of COVID-19 cases and deaths per million^1^^,^^2^.

		Brazil		Russia		India		China		South Africa	
**Total cases**		161,736,576 (6,272 339,803)		148,082,479 (31 49 941,735)		31, 508,918 (552 107,006)		6,292,359 (5 955 044,836)		6,946 (132 4512,836)	
**Total death**		3,195,763 (70 848,34)		2,696,193 (46 280,239)		373,295 (2 642,085)		19,96 (6 947,539)		1,708,204 (12 150,34)	
**COVID-19 incidence** ^**2**^		163,83		146,84		32,36		6,23		67,92	
**Case fatality ratio**		1.98%		1.81%		1.19%		0.55%		2.55%	
**COVID-19 death incidence**		3,24		2,67		0,38		0,02		1,73	

^1^ Results are given relative to the metrics of the World Bank geographical groupings of the BRICS country, which are shown in brackets, i.e. Brazil (Latin America and the Caribbean), Russia (European and Central Asia), India (South Asia), China (East Asia and Pacific), and South Africa (Sub-Sahara Africa) (31/10/2022).

^2^Incidence indicated as the number of cases per 1000 population.

**Table 2:** Comparative summary of COVID-19 statistics of BRICS relative to other international groupings (31/10/2022). Results are given relative to the metrics of the World Bank grouping classifications.

	BRICS	High-income countries	Low-income countries	Low-middle-income countries	Upper-middle-income countries
**Total cases**	>105.6M	>381.21M	>2.25M	>95.53M	>146.64M
**Total death**	>1.7M	>2.68M	>47.6M	>1.32M	>2.52M
**Total number of genomes from GISAID**	>0.5M	>11.7M	>0.028M	>0.47M	>0.78M

For BRICS, many of the pandemic milestones occurred in 2020, including the first recorded COVID-19 cases and deaths, community transmission, and disease dissemination within these countries, whereas 2021 and 2022 were distinguished by the emergence of genetic variants of SARS-CoV-2 (**Figs 1–5**). Overall, BRICS employed a combination of non-pharmaceutical interventions and a multifaceted vaccination rollout strategy to curb the spread of COVID-19. BRICS nations shared non-pharmaceutical interventions, including restricting movement, nationwide lockdowns at various stages, limiting social gatherings, and universal mask mandates were implemented to reduce respiratory transmission in public settings. In addition to these measures, genomic surveillance was employed across the BRICS nations to provide real-time data on the pandemic’s progression.

Below are summaries of the pandemic progression for each BRICS country over the study period, describing the epidemiological and pandemic milestones, non-pharmaceutical and pharmaceutical interventions, and insights gained from the genomic and surveillance data generated in each country.

### 1. Brazil

#### 1.1. Epidemiology and pandemic milestones

Brazil is the largest and most populated country in South America and has endured severe waves of COVID-19 (**Fig 1A**). Indeed, Brazil had the highest incidence of COVID-19 among the BRICS countries (**Table 1**). The first confirmed case in Brazil and South America was a traveler who displayed respiratory distress symptoms upon their return on 26 February 2020 [8] (**Fig 1B**). By 17 March 2020, the first reported fatality due to COVID-19 occurred, and on 13 March, the country exceeded >100 cases. Throughout March and April 2020, cases continued to increase, reaching > 100,000 by 3 May 2020 [1]. COVID-19-associated fatalities followed a similar pattern, as >100 deaths were recorded by April 2020, and in August, deaths exceeded >100,000.

Unlike global trends in 2020 and 2021, Brazil’s epidemiological curve did not peak and taper off as case numbers fluctuated. These observations most likely reflected resurgent infections. The highest mortalities recorded in the first two years of the pandemic occurred in April 2021. However, the most severe infectious wave occurred from January to April 2022. This infection wave peaked at 286,050 cases on 3 February 2022 [1], resulting in >8.25M cases and ~44,800 deaths. The next infectious wave was less severe and was recorded between late May and mid-August 2022, resulting in >3.36M cases and only ~2,700 deaths. The infectious waves were fueled by the Omicron strain and sub-lineages, which were thought to be more infectious yet resulted in fewer fatalities. During 2022, >12.5M cases and ~68,000 COVID-19-related deaths were reported in Brazil.

Seroprevalence analyses of specific cohorts highlighted the silent pervasiveness of the virus across Brazil. By the end of 2020, 83% of the population possessed antibodies against SARS-CoV-2 and highest seropositivity was found in studies conducted on the Northern region of Brazil, characterized by its low population density [27,28]. The causes for such a pattern are still under investigation but may be related to the undetected rise of the Gamma strain in the second semester of 2020 [29] and to the lower access to healthcare by rural populations [30–32]. Indigenous, low-income, homeless people, and other vulnerable populations also had higher rates of pre-vaccination seropositivity when compared to the general population [28,32–35].

#### 1.2. Non-pharmaceutical intervention

The federal government employed a combination of non-pharmaceutical interventions and a multifaceted vaccination rollout strategy to curb the spread of COVID-19. On 3 February 2020, the Brazilian Ministry of Health declared a state of National Health Emergency, encouraging social distancing, mask mandates, and restrictions on social gatherings [36]. The federal government did not issue a country-wide lockdown however, state and city governments independently directed and established social restrictions and social distancing measures. Consequently, lockdowns and restrictions were implemented heterogeneously across the country, with varying intensities and durations.

#### 1.3. Pharmaceutical intervention

Development and experimental trials of the ChAdOx1-S vaccine, which incorporates Oxford and AstraZeneca-based technologies, started in Brazil in June 2020 [37]. A month later, in-country trials for the Sinovac-CoronaVac vaccine were launched. Vaccination rollout strategies were implemented on 17 January 2021 [38]. Initial vaccination campaigns prioritized frontline workers, older adults (>60 years of age), and institutionalized adults with disabilities. The federal government would later incorporate the BNT162b2 Pfizer BioNTech and Ad26.COV2.S Janssen (Johnson and Johnson) vaccines. In mid-September 2021, the vaccination with booster doses was launched. By 31 October 2022, ~87% of the population had received at least the first dose of the vaccine, constituting >473M doses of vaccines [1].

#### 1.4. Genome sequencing and surveillance

Noteworthy pandemic milestones in Brazil included identifying VOCs and Variants of Interest (VOIs). These included the P.2 lineage that diverged from a B.1.1.28 progenitor. P2 circulated in high frequencies across the country [39–41] and was subsequently classified as the VOI Zeta by the World Health Organization (WHO). The P.1 lineage then displaced Zeta in late 2020 [39,40,42]. The P.1 lineage, subsequently named Gamma, was classified as a VOC and fueled an intense wave of COVID-19 infections in Brazil. The Gamma strain caused a high count of daily deaths that surpassed >3,100 per day in April 2021. During 2020, other lineages prevalent in Brazil included B.1, B.1.1.28, and B.1.1.33. The Gamma strain was first detected by Japan’s National Institute of Infectious Diseases (NIID) on 6 January 2021 from travelers returning from Brazil [39]. However, it was detected in Brazil less than a week later, on 12 January 2021. Retrospective genome surveillance reports that the Gamma strain was sampled from COVID-19 patients as early as 7 August 2020, suggesting it circulated at a reduced frequency for months in Brazil before displacing the P.2 strain. The VOC Delta was introduced to Brazil in May 2021 [43] and rapidly displaced Gamma; with Delta subsequently outcompeted by the VOC Omicron BA.1, detected in Brazil on 30 November 2021, BA.2 on 14 January, BA.4 and BA.5 on 18 May 2022 and 7 June 2022, respectively (**Fig 1B**). The BA.4 and BA.5 lineages circulated in notable frequencies from June to October 2022.

**Fig 6:**
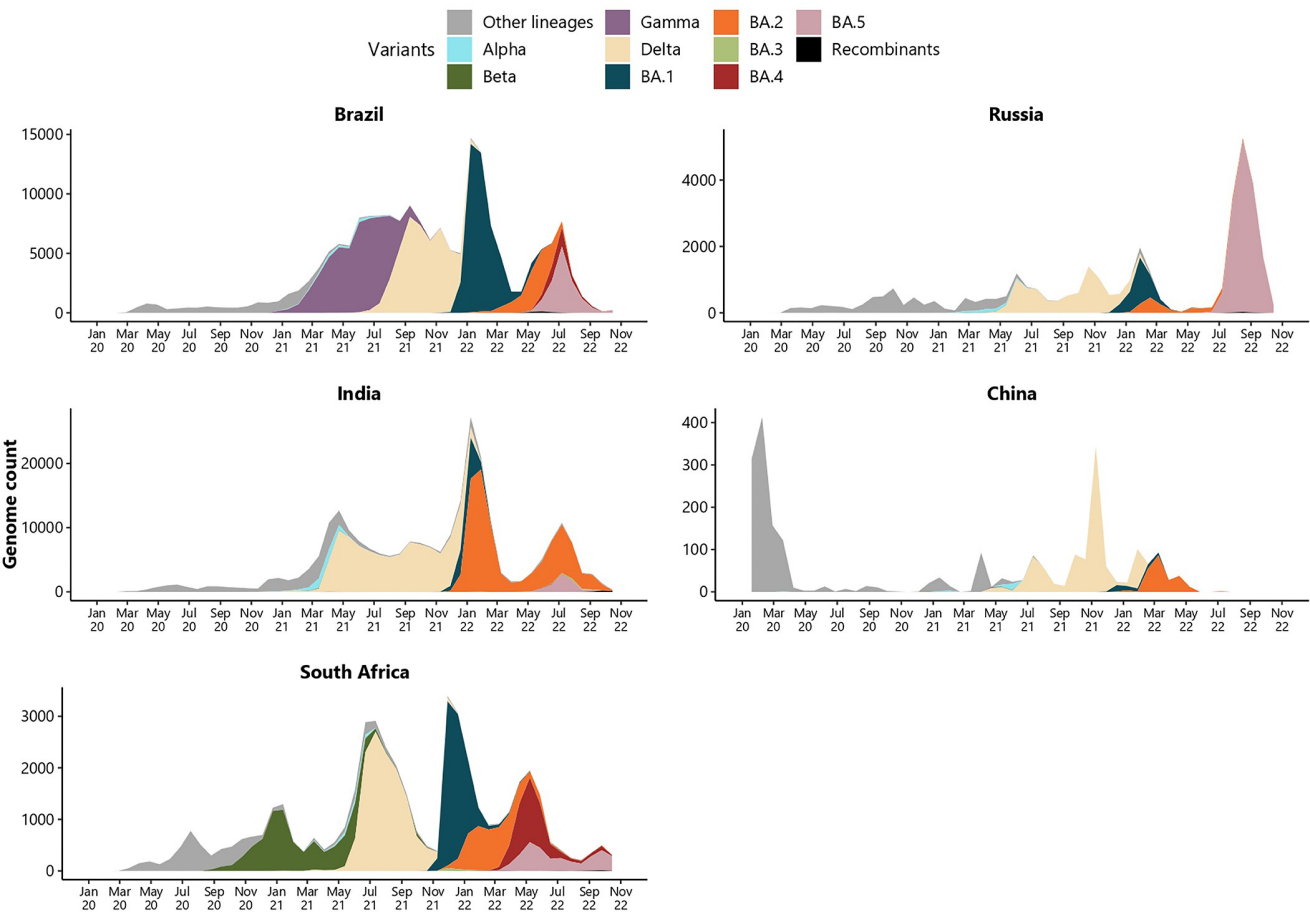
Schematic representation of the changes in the genetic compositions of SARS-CoV-2 lineages recorded for BRICS countries for 1 January 2020 to 31 October 2022.

The epidemiological data for Brazil shows a unique pattern in case numbers and deaths (**Fig 1A**). In 2020, there was an increase in cases from March 2020 to early November 2020, with the peak occurring in August 2020. Gamma’s rapid displacement of Zeta and other lineages in December 2020 prompted a new surge in cases. The lineage-through-time plots (**Fig 6**) indicate that Brazil was the only BRICS country where the Gamma strain circulated at high prevalence.

This second infectious wave continued to increase until April 2021 and steadily decreased until January 2022. In August 2021, the Delta strain displaced the circulating Gamma strain (**Fig 6**), fueling a third infectious wave. Genomic data suggests that the fourth peak during this period was driven by the Omicron BA.1 strain and caused an unprecedented increase of 6.5M cases in January 2022 [1]. The BA.2 Omicron strain circulated at high frequencies from March to July of 2022 until it was displaced by the BA.4 and BA.5 strains of Omicron. Amongst the circulating VOCs and VOIs sampled, the Beta, Lambda, Mu, and recombinant strains did not circulate in significant frequencies (**Fig 6**).

### 2. Russia

#### 2.1. Epidemiology and pandemic milestones

Russia is the largest country in size, one of the most populous countries in Europe, and has suffered extensive waves of COVID-19. Amongst the BRICS countries, Russia had the second-highest incidence of COVID-19 (**Table 1**). The first cases of COVID-19 were reported as early as 31 January 2020 **(Fig 2B)** [44]. On 2 March 2020, a traveler returning from Italy to Moscow was diagnosed with COVID-19 [45], and on the 19th of March, the first fatality in Russia occurred, having resulted from community transmission [46]. Cases increased from 10,000 to >1M between April and 2 September 2020 [1]. In total, >21.1M cases and >382,000 deaths were reported [1]. The data on the reported cases and deaths in Russia has been widely debated based on extensive excess mortality statistics, suggesting that these reported cases and deaths may remain underreported [25].

The first infection wave occurred in May 2020, resulting in >4M cases and >17,000 deaths (**Fig 2B**). This was followed by a second wave that peaked in December 2020, resulting in >3M cases and 80,000 deaths. Two more infectious waves were recorded in 2021, the first of which peaked in July and the second in November. This resulted in >2.5M cases and >100 000 deaths, and >3.6M cases and >130 000 deaths, respectively. As in Brazil, the most severe wave occurred from January to April 2022, resulting in >7M cases and >49,000 deaths. The final infectious wave recorded during the study period started in early July 2022. This wave continued until October 2022, resulting in >3.1M cases and >10,500 deaths. However, following the global trend, the Omicron VOC and its descendent lineages fueled infectious waves with a resultant lower mortality rate than previous waves.

Compared to other European countries, Russia was the most severely affected by COVID-19 between early May 2020 and late July 2020 [1]; by October 2022, it was ranked the country with the 5th highest cases on the continent and 10th highest globally [1]. Additionally, Russia recorded the highest number of COVID-19 deaths in Europe and ranked 4th highest globally, lagging behind the United States, Brazil, and India, all countries with larger populations [1]. Most deaths took place between March and June 2022, with the deadliest waves being fueled by the Delta and the Omicron BA.2 strains (Fig 6). The onset of epidemiological waves in Russia generally mirrored that in Europe but lagged a few months behind, consistent with the delayed introduction through Europe of the earliest SARS-CoV-2 lineages [47,48]. The exception was the Omicron wave, from March to August 2022, which was not pronounced in Russia for unclear reasons [49].

#### 2.2. Non-pharmaceutical intervention

The Russian government implemented disease intervention strategies early in the first quarter of 2020. These included restricted access across its Chinese border and domestic non-pharmaceutical interventions of lockdowns, mask mandates, and restriction of social movement and travel **(Fig 2B)** [50]. Mask mandates were implemented on a region-specific basis and were directed by local governmental authorities. The first was introduced on 12 March 2020 and served as an essential pandemic intervention strategy in highly populated regions such as Moscow and St. Petersburg. No lockdowns and restriction of movement mandates were implemented in Russia during 2022. The nationwide mask mandate was lifted on 2 July 2022. However, the lifting of these mandates was replaced by region-specific mandates on mask usage directed by local governmental regulatory authorities [51].

#### 2.3. Pharmaceutical intervention

Russia was one of the first countries in the world to implement mass vaccination campaigns [52,53]. The Sputnik V vaccine, formally registered as Gam-COVID-Vac, was deployed on 27 November 2020, and the formal vaccination campaign was deployed on 5 December 2020. By 31 October 2022, >180.3M doses were administered [1].

#### 2.4. Genome sequencing and surveillance

Analyses of genome sequences showed that during 2020, a combination of B.1 ancestral lineages circulated in Russia. By January 2021, both Alpha and Beta strains had circulated in Russian populations (Fig 2B) [54]; however, these strains had not become predominant during the progression of the pandemic. By June 2021, the Delta strain had increased in prevalence and displaced both Alpha and Beta strains, rapidly becoming the dominant strain (Fig 6). In fact, by January 2022, >50% of SARS-CoV-2 assemblies were assigned to the Delta lineage (n = 7,253). Interestingly, >90% of Russia’s Delta cases probably descended from a single early introduction of this strain [47,54]. Only a single Gamma strain sequence from Russia has been submitted to the GISAID repository, suggesting that this strain did not circulate in high frequencies within Russian populations. Following the global trends and those observed in other BRICS countries, the Omicron lineages increased in prevalence since its first detection on 7 December 2021 and ultimately replaced other SARS-CoV-2 lineages. The BA.2 sub-lineage of the Omicron strain was sampled as early as January 2022, BA.5 19 February 2022, and the first BA.4 variant was collected on 25 May 2022. BA.2 was the dominating strain from January to July 2022, after which the BA.5 strain displaced it.

### 3. India

#### 3.1. Epidemiology and pandemic milestones

Globally, regions with high population densities experienced severe outbreaks, especially during the onset of the pandemic. However, India, the world’s second most populous country, reflected a slow pandemic progression during the first semester of 2020 (**Fig 3A**). The country subsequently reported more COVID-19 cases and deaths than other BRICS countries [1]. In fact, by 31 October 2022, the severity of the pandemic in India (n = 44,654,638 cases; n = 529,077 deaths) was surpassed only by that of the United States of America (n = 97,505,662 cases; n = 1,070,622 deaths) [1].

The first cases were reported in the state of Kerala on 30 January 2020 by a student returning from Wuhan, China [55] (**Fig 3B**). During the first three years of the pandemic, India experienced four waves of infection (**Fig 3A**). The first occurred between May 2020 and February 2021, and peaked at ~98,000 cases on 16 September. A second, more severe wave of infection (**Fig 3**), peaked on 6 May 2021 with 414,188 cases. The second wave resulted in >23.9M cases and >320,000 deaths. The third wave peaked on 14 January 2022 and reached a pandemic milestone with the highest count in reported cases (n = 533,035). This wave resulted in ~8M cases and ~33,000 deaths [1]. The fourth infectious wave was the least severe and occurred between mid-June and mid-September of 2022, resulting in ~1.27M cases and ~3,000 deaths.

In India, seroprevalence was observed to increase by 3-fold between the first (March-November 2020) and second (March-May 2021) pandemic wave. A steep rise in antibody titre was observed with Delta-VOC infections in the second COVID-19 wave, while it showed substantial increase with vaccination throughout the country. Seroprevalence was predominant across urban areas as compared to the rural areas because of differential exposures. Various serosurveillance studies revealed that a large proportion of the population had undergone an asymptomatic infection [56–58].

#### 3.2. Non-pharmaceutical intervention

Non-pharmaceutical intervention strategies were implemented as early as January 2020 [55]. These included implementing contact tracing strategies, thermal screening of travelers, mask-wearing mandates, and closing educational and other public institutes. These restrictions included the Janta Curfew, which restricted freedom of movement for 14 hours a day, implemented on 22 March 2020. On 25 March, a nationwide lockdown was implemented [59]. The Indian territories were demarcated into green, red, and orange zones based on disease transmission risks [60]. Green constituted the most minor at-risk territories, and red was the most severely affected. Restrictions were gradually eased and phased out based on the severity of the pandemic. In March 2020, one of the largest global testing databases was launched [60,61]. On 15 April 2020, TrueNAT, the first point-of-care molecular diagnostic COVID-19 test in the world, was approved by The Indian Council of Medical Research (ICMR), which was subsequently approved by the WHO [62]. In May 2020, a mass repatriation strategy was launched, named the "Vande Bharat Mission" [63]. This mission aimed to assist distressed Indian citizens worldwide in returning to India.

#### 3.3. Pharmaceutical intervention

The government began nationwide administration of COVID-19 vaccines on 16 January 2021; roughly a year later >41.4M vaccine doses were administered, which increased to >950M doses by 31 October 2022 [1]. During the vaccination rollout, both Oxford–AstraZeneca (developed under license by Serum Institute of India, under the trade name Covishield) and Covaxin (developed in India by Bharat Biotech) vaccines were approved for use. On 13 April 2021, permission for restricted use of the Sputnik V vaccine (produced by the Serum Institute of India) was granted in emergencies by the Drugs Controller General of India [64].

#### 3.4. Genome sequencing and surveillance

Together with non-pharmaceutical and vaccination rollouts, pandemic intervention efforts were complemented and guided by large-scale collaborative genome surveillance initiatives. These initiatives included the Pan India 1000 SARS-CoV-2 genomic consortium launched in 2020 [65]. The Pan India 1000 SARS-CoV-2 genome genomic consortium involved numerous stakeholders and institutes associated with the Indian governmental Department of Biotechnology (DBT). It was coordinated by the National Institute of Biomedical Genomics (NIBMG) in West Bengal. Similarly, the Pan-India network, known as the Indian SARS-CoV-2 Genomics Consortium (INSACOG) [66], was launched on 28 December 2020. This network involved the concerted efforts from the Ministry of Health and Family Welfare (MoHFW), DBT, Council for Scientific and Industrial Research (CSIR), Indian Council of Medical Research (ICMR), National Centre for Disease Control (NCDC), Central Surveillance Unit (CSU), and the Integrated Disease Surveillance Programme (IDSP). The data generated from these consortiums proved invaluable to monitor and detect SARS-CoV-2 variants and to investigate the pandemic progression across India [66].

The B. 1 ancestral lineages fueled the first wave and were not due to distinct VOCs or VOIs (**Fig 6**). The Alpha strain was detected on 29 December 2020, and the Beta strain on 26 March 2021 however, these strains did not circulate at high prevalence. The second wave was fueled by the Delta VOC, which first emerged in India [67]. An increased circulation of the Delta strain was reported in Maharashtra on 31 March 2021 [68–70]. Community transmission of the Delta strain resulted in the most severe infectious wave, in terms of case numbers and deaths, recorded to date (**Fig 3A**). This infectious wave was associated with alarming reports of increased community transmission and nationwide and localized outbreaks. The first case of Omicron BA.1 was detected from a sample isolated on 28 November 2021 (and submitted to GISAID in early December 2021), which was from a person with an international travel history [64]. The Delta and Omicron BA.1 and BA.2 strains were prevalent in the third infectious wave that was recorded in January 2022 (**Fig 6**). This wave peaked on 14 January 2022 and reached a pandemic milestone with India’s highest reported cases (n = 533,035). This wave resulted in ~8M cases and ~33,000 deaths [1]. The BA.2 Omicron strain dominated this wave, resulting in fewer COVID-19 deaths. The dampened death rate is thought to be due to the roll-out of vaccination campaigns. The genetic composition of the fourth wave included BA.2 and BA.5 Omicron variants. The VOCs Beta, Gamma, and the VOI, Mu, did not reach significant prevalence in India. The total number of genomes produced in India has increased noticeably since December 2020 (**Fig 3**), and >313,000 whole genome sequences have been deposited on GISAID.

### 4. China

4.1. Epidemiology and pandemic milestones

The COVID-19 pandemic emerged in China, the most populous country in the world **(Fig 4)** [71,72]. The first outbreak was reported in Wuhan, Hubei Province, in early December 2019. On 27 December 2019, Hubei Provincial Hospital of Integrated Chinese and Western Medicine reported clusters of cases of pneumonia of unknown cause to the Wuhan Jiangshan Centre for Disease Prevention and Control [71,72]. COVID-19 has since spread to all Chinese provinces [72] and underlies the emergence of the first of many global infectious waves.

Initial investigations aimed to determine the origin and causative agent underlying the pneumonia outbreaks [73]. To this end, epidemiological investigations characterized and reported the first strain of the novel coronavirus. Chinese researchers confirmed that the underlying pathogen was the novel severe acute respiratory syndrome coronavirus 2, or SARS-CoV-2, causing the coronavirus disease, now globally known as COVID-19. This outbreak continued until late March 2020, resulting in >80,000 cases and >3,000 deaths [1]. This outbreak peaked on 13 February 2020 [71]. China was the first to control the mass outbreak of COVID-19 endemic transmission. In fact, during the first two years of the pandemic, China reported 610 days of <100 new cases [1] and was also the BRICS country with the lowest case incidence and lowest case fatality ratio (0.55%; **Table 1**).

Seminal research from Chinese institutes paved the way for molecular, genomic, and epidemiological investigations of the virus [74–77]. In early January 2020, the first whole-genome sequence of SARS-CoV-2 was generated and released to publicly available databases such as GISAID and NCBI, thus providing immediate and free access to the global scientific community [74–77]. This led to the successful design and production of the first SARS-CoV-2 test kits, which were optimized for polymerase chain reaction (PCR) analyses [78]. These strides facilitated countries internationally to implement rapid testing and efficiently adopt disease intervention strategies. Moreover, the first genome assembly laid the foundation for global investigation of SARS-CoV-2 evolution and genetic divergence and determining key aspects of its phylogeography. However, despite these seminal contributions, the WHO recently raised concerns about data transparency, encouraging the Chinese CDC to contribute its information to the scientific community to verify the accuracy of the data reported by the Chinese government [79,80].

China recorded only a single epidemiological wave in 2020 (**Fig 4A**); however, the total number of cases during this time was thought to be underestimated [80]. A study evaluated the seroprevalence of 17,368 individuals in Wuhan and other geographic regions in China during the period of March 9, 2020 to April 10, 2020 and found seropositivity in Wuhan ranged from 3.2% to 3.8%, and progressively decreased in other cities as the distance from Wuhan increased [81]. For China, smaller outbreaks were reported in January 2021, resulting in 2,475 cases and zero deaths. More severe outbreaks, with a higher number of reported cases (**Fig 5**) [1], occurred during 2022 and were fueled by the Delta and BA.1 and BA.5 Omicron strains (**Fig 6**). The first of which resulted in >758,000 cases and 590 deaths, lasting from March 2022 to the end of May 2022. The second mass outbreak occurred between July and was considered ongoing by 31 October 2022, resulting in ~144,000 cases and ~20 deaths. Despite the relatively higher number of cases reported in 2022, China reported substantially fewer COVID-19 cases and deaths among the BRICS countries. Only ~120,000 cases and > 4,600 deaths were recorded by 31 January 2022, which increased to 956,492 cases and 5,549 deaths by 31 October 2022.

#### 4.2. Non-pharmaceutical intervention

Following the reports of the first outbreak, a range of restrictive measures and disease intervention strategies were imposed, constituting the first of their kind [82]. These included converting stadiums and exhibition centres into 16 temporary treatment centres providing >14,000 beds. Two emergency hospitals that specialized in the treatment of COVID-19 were provisioned during the outbreak and had >1,000 beds per hospital. On 31 December 2019, face masks were implemented in Wuhan. On 1 January, the Wuhan Huanan Seafood Wholesale Market was closed [72,83]. This market was the suspected source of infection during the outbreak. On 23 January 2020, the city of Wuhan was placed under lockdown [72,84]. This included closing all city entry and access points, such as airports, railway systems, roads, and waterways. All public gatherings were cancelled. Cultural and entertainment venues were closed, and the opening of schools and universities was postponed. All COVID-19 patients were placed under mandatory quarantine, where all inbound travelers were required to quarantine under medical observation for 14 days, followed by an additional seven days of home observation. These restrictions on freedom of movement and limited social interaction strategies were the first of their kind when similar strategies were adopted globally [72,84–86].

Following the first report of the Omicron variant from Chinese patients, isolated on 27 November 2021 in Hong Kong and 9 December 2021 in mainland China [87]. The first GISAID submission, however, was sampled on the 30^th^ of December. In intervention to detecting the highly transmissible Omicron variant, regional lockdowns were implemented based on the risk level of community transmission, allowing for targeted disease intervention strategies. This was also referred to as “silent management” [88] due to the affected areas becoming smaller as the outbreaks subsided. From August 2021 until December 2022 [86], this was also called the “Dynamic COVID-Zero strategy”, reflecting the alteration in severity and duration of pandemic intervention strategies implemented to hamper the economic and social side effects of harsh lockdowns. As the pandemic progressed, the time for medical observation in isolation was reduced from 21 days to 14 days [89].

#### 4.3. Pharmaceutical intervention

China adopted a rigorous vaccination rollout strategy in July 2020 [90]. It prioritized front-line workers and volunteers. By the end of November 2020, >1.5M doses were administered [1]. From 1 December 2020, the vaccine was rolled out for workers involved with cold chain coordination, customs and border inspection, medical and disease control, public transport, and personnel. By 31 October 2022, >3.4 B doses of COVID-19 vaccines were administered [1], using COVID-19 vaccines that the National Medical Products Administration of China approved. The Sinopharm BIBP was developed by Sinopharm’s Beijing Institute of Biological Products and Sinopharm WIBP from Sinopharm’s Wuhan Institute of Biological Products. Additionally, Sinovac’s CoronaVac was produced by Sinovac Biotech. CanSino Biologics created Convidecia, and ZF2001, also known as Zifivax, was developed by Anhui Zhifei Longcom in collaboration with the Chinese Academy of Sciences [1].

#### 4.4. Genome sequencing and surveillance

Like global trends, the first outbreak did not consist of dominating VOCs or VOIs. During the progression of the pandemic timeline, all VOCs and descending lineages were detected amongst Chinese residents, albeit in sparse numbers. The Delta strain circulated until March 2022, when it became displaced by the Omicron BA.1 strain, which too became displaced by the Omicron BA.2 strain **(Fig 6)**. Genome surveillance data deposited by China suggests that amongst the VOCs and VOIs investigated in this study, the Beta, Lambda, Gamma, Omicron BA.4 and BA.5 strains, and Mu strains did not circulate in high frequencies in China during the study period.

### 5. South Africa

#### 5.1. Epidemiology and pandemic milestones

The first case of COVID-19 in South Africa was reported on 5 March 2020 from an infected traveler returning from Italy, and on 27 March 2020, the first South African succumbed to COVID-19 **(Fig 5)** [1]. The first case was reported in KwaZulu-Natal and was soon followed by community transmission and rapidly spread to all nine provinces [91]. South Africa has reported >4M cases and >102,000 deaths (31 October 2022) [1] **(Fig 5)**. The three most severely affected provinces included Gauteng, KwaZulu-Natal, and the Western Cape [92], which were also amongst the most populous.

South Africa has endured five waves of COVID-19 infections **(Fig 5A)** [1]. The first occurred from May to September 2020, resulting in >600,000 cases and >14,000 deaths. This wave peaked on 19 July 2020 at 13,499 cases. The second wave occurred between November 2020 and March 2021 and peaked on 8 January 2021, with 21,980 cases recorded in a single day. This wave resulted in 775,000 cases and > 28,000 deaths. The third wave occurred from May until October 2021, peaking on 8 July 2021 at 22,910 cases. This wave resulted in >1.3M cases and > 36,000 deaths. The fourth wave lasted from November 2021 to February 2022, resulting in >724,000 cases and >9,700 deaths. The fourth wave peaked on 12 December 2021 at 37,875 cases, exceeding previous records. The final wave was observed from April 2022 to mid-June 2022 and reported the lowest counts of cases and deaths, >250,600 and >1,400, respectively. Unlike the remainder of BRICS, South Africa did not experience high case numbers for an extended period from July to October 2022. South Africa recorded the third-highest incidence of COVID-19 among BRICS members. South Africa had the highest case fatality ratio amongst BRICS countries (2.55%; **Table 1**). However, studies on seroprevalence suggested that many cases were asymptomatic, where seropositivity was more prevalent amongst lower-income participants, emphasizing that the actual burden of the pandemic may have been underestimated and may have disproportionately affected economically disadvantaged communities [93].

### 5.2. Non-pharmaceutical intervention

The epidemic intervention was launched in January 2020 [94]. The emergency management model incorporated a coordinated intervention involving sectors collaborating to establish the Incidence Management System (IMS) [95]. The IMS included the expertise of existing governing infrastructures to optimize local and national governance to facilitate pandemic intervention. This expertise included risk communication, community engagement, securing medical supplies and facility readiness, case management, tracking and tracing, and coordinating emergency medical services [95]. On 15 March 2020, a national state of disaster was declared, followed by the inauguration of the South African National Coronavirus Command Council [96]. Schools were directed to close on 18 March 2020. A national lockdown was implemented on 27 March 2020. This social and economic lockdown was initially instated for three weeks but was extended until 30 April 2020 [97]. A five-phase lockdown was thereafter incorporated. Level five was reserved for periods of extreme levels of community transmission, hospital admissions, and daily cases. Level one was instituted when these pandemic indicators were at reduced risk. These lockdown regulations remained instated until April 2022, accounting for 750 days of lockdown [98].

### 5.3. Pharmaceutical intervention

The vaccination campaign was initiated on 17 February 2021. The vaccine brands approved for mass vaccination campaigns included Janssen (Johnson & Johnson) and Pfizer-BioNTech [98]. The first of the three-phased vaccination strategies was directed at the vaccination of healthcare workers. Phase two prioritized demographics of citizens registered as essential workers, persons in congregate settings, individuals 60 years and older, those with comorbidities, and individuals deemed more vulnerable to severe infection [99]. Phase three catered for the vaccination of individuals 18 years and older and was initiated in August 2021. As of 31 January 2022, a year since the initial launch of the vaccination campaign, ~30M doses were administered, resulting in >16M fully vaccinated individuals from >3,300 vaccination points across the country [1]. By 31 October 2022, ~38M doses and >19.46M people were fully vaccinated, constituting a third of the population, which remains below the 70% target. South Africa represents the least vaccinated country in BRICS, which was thought to be due to widespread vaccine hesitancy [100]. Concerns about the emergence of VOIs and VOCs originating from southern Africa remain high as it represents populations with high frequencies of immunocompromised individuals that remain infected for more extended periods, low vaccination coverage, and strained public health care systems [101].

### 5.4. Genome sequencing and surveillance

Pandemic milestones in South Africa included the detection and reporting of circulating VOCs. The most notable included the Beta and the Omicron strains (BA.1 and BA.2) in December 2020 and November 2021, respectively [91,102]. These two VOCs fueled regional and global infectious waves during 2021 and 2022. Genomic data indicated that all VOC strains, except for the Gamma strain, circulated in notable frequencies in South Africa during the first two years of the pandemic **(Fig 5A).** Comparable to other BRICS countries, B.1 associated ancestral lineages circulated during the first wave. However, the Beta strain dominated the second wave, and the Delta strain the third. However, notable frequencies of the Alpha and Beta strains were detected during the third wave. The Omicron strain fueled the fourth wave and included 4,170 submissions to the GISAID platform by 31 January 2022. The Delta strain remained most prevalent until November 2021. Since November 2021, the Omicron strain BA.1 and its genetic descendants, BA.2, BA.4, BA.4, and BA.5, have dominated the majority of all circulating VOCs in South Africa. Of these epidemiological waves, the second recorded the highest number of deaths, and the fifth wave of infection, the least.

### B. Genome surveillance: SARS-CoV-2 genomic and epidemiology dynamics in BRICS countries

BRICS nations and the NGS-BRICS contributed to the real-time surveillance of SARS-CoV-2 to monitor the emergence of new variants (**Tables 1 and 2**) [39,40,69,91,102,103]. To this end, BRICS generated >500,000 SARS-CoV-2 whole-genome sequences (**Table 2**; 31 October 2022). Of these sequences, India generated ~313,000, Brazil ~189,000, Russia ~35,000, and South Africa ~47,000, whilst China (mainland China) deposited ~2,700 whole-genome sequences to the GISAID database.

**Fig 7:**
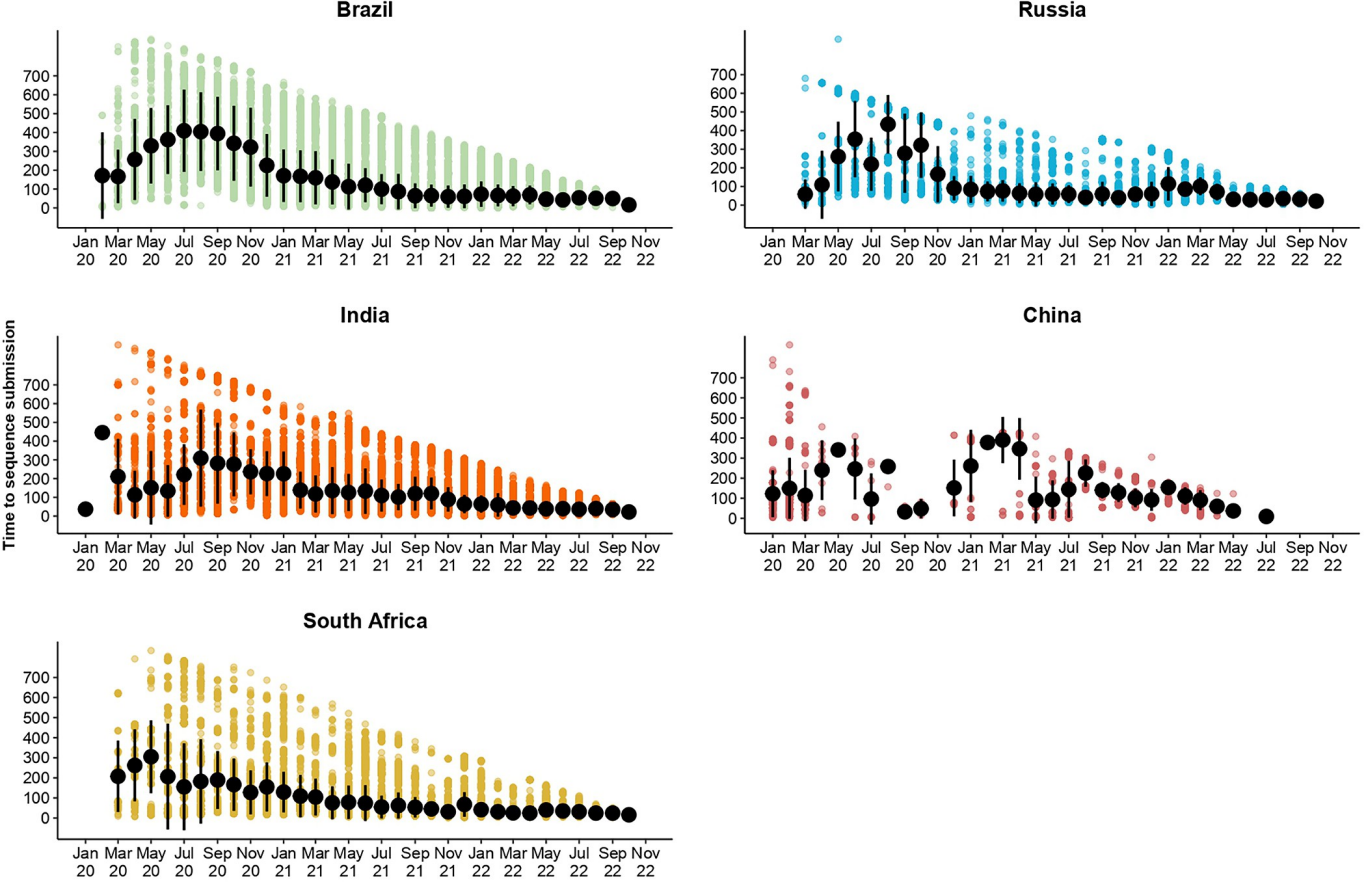
Graphical summary of the average number of days (+- SD) from sample collection to sequence submission to GISAID for the BRICS countries. The results are illustrated per month recorded for BRICS countries from 1 January 2020 to 31 October 2022.

BRICS countries produced notable improvements in the total count of genomes produced over time as the pandemic progressed. Brazil deposited ~6,700 genomes in 2020, ~93,200 in 2021, and ~96,000 in 2022. Russia generated ~4,400 (2020), ~9,700 (2021), and ~23,000 assemblies (2022). India generated ~12,000 (2020), ~94,500 (2021), and ~180,000 (2022) assemblies. China generated ~1,000 (2020), ~400 (2021), and ~1,200 (2022) genome assemblies. South Africa generated ~6,500 (2020), ~22,000 (2021), and ~21,000 (2022) assemblies. Moreover, the average number of days from sample collection to sequence submission to GISAID followed similar trends (**Fig 7**). Such advances in turnaround times are essential for real-time data availability to enable informed BRICS pandemic interventions. For BRICS, the total number of genomes submitted to GISAID reduced notably following a reduction in the case of numbers (**Figs 1–5**). This observation follows global trends where reduced case numbers corresponded with fewer genome assemblies generated as fewer samples were available for genome surveillance [104,105].

The NGS-BRICS countries generated ~534,000 genome assemblies. In comparison, high-income countries (according to the WHO classifications) generated and deposited 11.7M SARS-CoV-2 genome assemblies. Upper-middle-income countries deposited 0.78M genomes, whereas low-income and low-to-middle-income countries generated 0.028M and 0.47M, respectively (**Table 2**). These statistics reflected the lack of infrastructure to support genome surveillance in BRICS countries during the onset of the pandemic [106]. However, as the pandemic progressed, BRICS investment in genome surveillance equipment and laboratory and bioinformatic expertise increased, and broader availability and accessibility to reagents facilitated BRICS genome surveillance.

**Fig 8:**
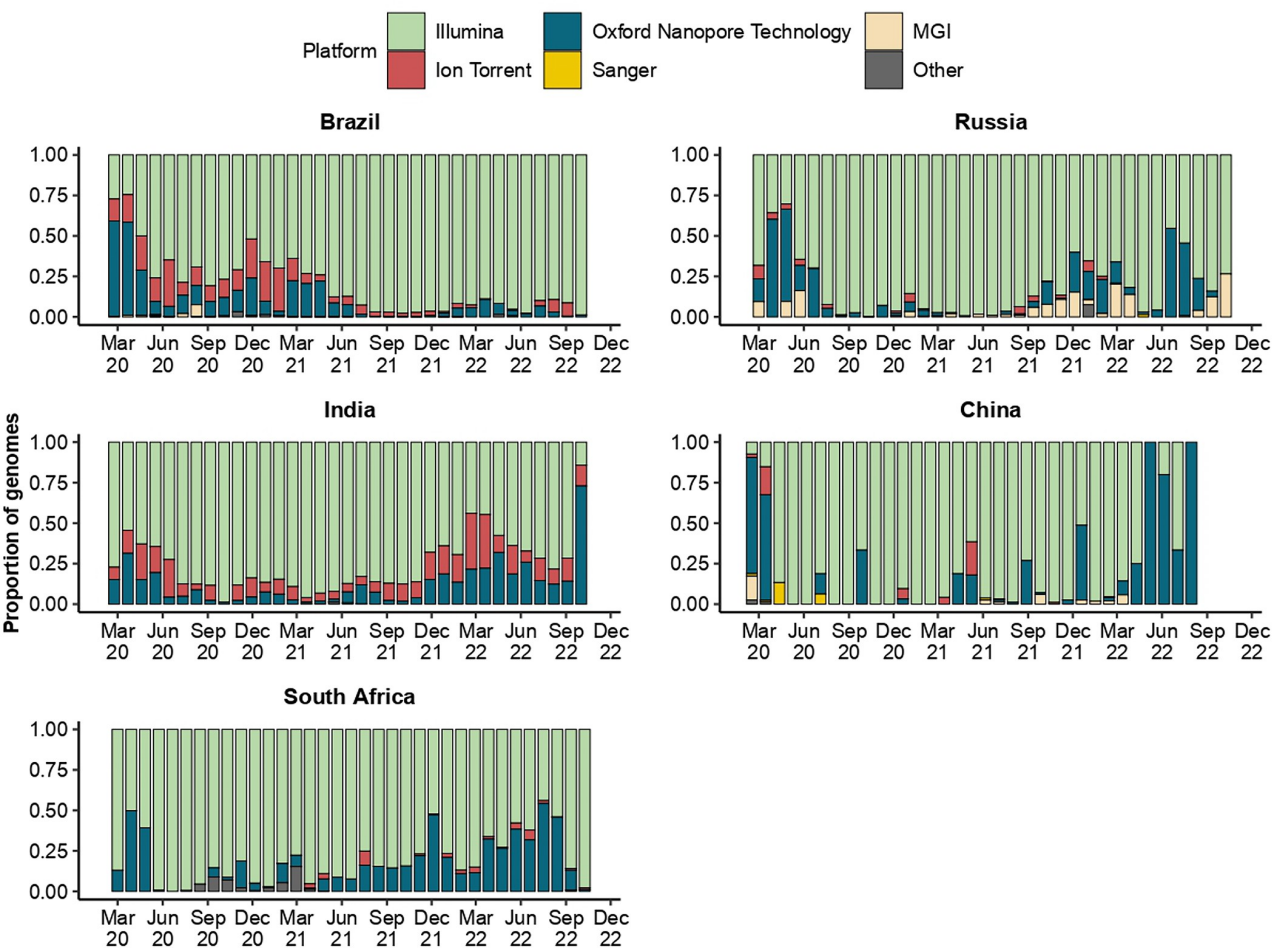
Illustration of the total proportions of sequencing platforms used by BRICS countries to produce SARS-CoV-2 whole-genome sequences submitted to GISAID (1 January 2020 to 31 October 2022).

The whole genomes generated by BRICS were performed on five sequencing platforms (**Fig 8**). These included Illumina (n = 356,330), Oxford Nanopore Technologies (ONT; n = 42,759), Ion Torrent (n = 27,229), MGI (n = 2,345), and Sanger (n = 104). Of these platforms, a considerable proportion of genomes were generated using the Illumina platform (61%), followed by ONT (7%). The Ion Torrent (5%), MGI (0.4%), and Sanger (0.01%) platforms were used the least. Only Brazil, China, and Russia indicated the usage of the MGI platform, particularly during their respective first infectious waves that occurred in 2020. However, Russia used the MGI platforms throughout the progression of the pandemic. All countries relied on Illumina platforms for genome surveillance; however, during the second half of 2022, Chinese institutes favored using ONT platforms. Moreover, only China used the Sanger sequencing platform among the BRICS countries. The use of these five platforms most likely reflected the availability of existing infrastructure during the onset of the pandemic; however, investment in ONT and Illumina platforms became more available in BRICS countries as the pandemic progressed.

### C. Summary of BRICS pandemic interventions and genomic surveillance

This study analyzed BRICS countries’ COVID-19 interventions in relation to the genomic and epidemiological milestones accrued throughout the height of the COVID-19 pandemic via comparative assessments. These analyses identified national approaches from China’s early containment strategies, including the first-of-its-kind lockdowns and fever clinics and mask-wearing, and described Brazil’s interventions during extended infection periods. Genomic surveillance contributions included the identification of Beta and Omicron variants in South Africa, Delta in India, Gamma that originated in Brazil, and the initial SARS-CoV-2 genome sequence generated in China, techniques that were shortly used globally. Progress in the development of response strategies was exemplified by Russia’s transition from broad lockdowns to targeted interventions. BRICS-specific challenges, such as India’s high-density population management, prompted solutions like zone-based restrictions. Our analysis emphasizes integrating diverse approaches in global health intervention strategies.

## Discussion

BRICS genome surveillance and disease intervention strategies directed important aspects of the global health landscape during the COVID-19 pandemic. This is of particular significance as BRICS nations encompass almost half the world’s population [1], many of which constitute resource-limited settings. Due to the improvements in NGS-BRICS high-throughput genome surveillance capacity and the increased availability of both experimental and bioinformatic expertise, BRICS optimized genome surveillance capacity as the pandemic progressed (**Figs 1–5** and **Figs 7 and 8)** [107–110]. This, in turn, facilitated the detection of numerous BRICS-emerging VOCs and VOIs [39,40,69,91,102,103]. These improvements further facilitated their genome surveillance initiatives, data generation, and collaboration with other developing-, low- and middle-income countries [111]. Despite the increased capacity, BRICS deposited only a small share (4.28%) of the total whole-genome sequences produced globally, and high-income groupings deposited 85.3% [24].

BRICS has endured high case numbers and deaths during the pandemic; many have the highest number of cases for their respective regions and continents, and BRICS remained vulnerable to the introduction of newly emerging variants. The introduction of VOCs and the resulting increase in case numbers occurred due to the high level of travel and the movement of infected individuals across country borders. These high travel volumes reflect the social and economic importance of BRICS countries, constituting their respective continents’ economic and travel hubs. BRICS experience high travel volumes for business, import and export, tourism, and political or civil refuge purposes [112]. Indeed, globally, the initial SARS-CoV-2 strain, and its descendant variants were introduced due to international travel [1,45]. BRICS countries are also highly populated. More populous countries, where large numbers of people reside in close proximity, remain more vulnerable to severe pandemic waves and higher counts of deaths due to overburdened healthcare infrastructures [2,3]. Moreover, access to large-scale vaccine availability may remain challenging for developing countries [3]. Such concerns are particularly evident in rural regions with limited access to adequate health services, which underlie vaccine inequality concerns [113].

Epidemiological analyses showed that the frequency and timing of infectious waves were country-specific (**Fig 5**). A combination of co-circulating VOCs was often recorded amongst BRICS countries. Viral lineage displacement events fueled renewed pandemic waves. The detection and characterization of VOCs served as an early warning system to alert public health authorities of upcoming surges of infections (**Figs 1–7**). Moreover, the Beta and Omicron BA.1 strains reported from southern African research institutes [91,102] displaced their pandemic predecessors more rapidly and earlier in the pandemic timeframe than in other BRICS countries where these VOCs were introduced (**Fig 6**). The Gamma strain displaced its genetic predecessors in Brazil. In contrast, Delta swiftly displaced other circulating lineages occurring in India. This finding most likely reflected the genetic mutations in the genomic regions underlying viral dynamics, such as enhanced transmissibility and immunity escape, providing a competitive advantage over previously circulating lineages [102]. Moreover, retrospective analyses of deposited data often inferred that many earlier VOCs circulated undetected in their countries of putative origin. However, due to enhancements in bioinformatic and experimental expertise, later strains and derived lineages, such as that observed for the later emerging Omicron lineages, were detected much more readily [102].

Molecular epidemiology data showed that four of the five VOCs reported during the first two years of the pandemic underlie severe infectious waves in BRICS, while other VOIs were less prevalent. Unlike the VOCs, the VOIs Lambda and Mu did not circulate at meaningful frequencies. However, they were prevalent in Peru, Argentina, Chile, Ecuador, and Colombia in the second half of 2021 [40,114]. The VOCs, bar the Alpha strain, were reported in high frequencies by BRICS (**Fig 6**). The Alpha strain underlined extensive waves of infection in the United Kingdom, many European countries, and North America [115,116]. Amongst BRICS countries, a high frequency of circulating Gamma strain was unique to Brazil, where it first emerged. The Omicron strains disseminated globally and remained prevalent in BRICS countries from late 2021 and continued throughout 2022. Distinctive prevalence in circulating lineages throughout the pandemic supported the view that genome surveillance provided crucial information on the country- or regions-specific level, directing health interventions tailored to the dynamics of currently circulating variants.

This work represents the first of its kind to describe the output generated by BRICS that places pandemic interventions in relation to their epidemiological and genomic milestones. BRICS contributed vital scientific data as the pandemic progressed globally. This included the first disease report, seminal work on the etiology underlying the COVID-19 pandemic, and the first full-length sequence of SARS-CoV-2 [72,73]. Moreover, BRICS nations first detected and reported many of the VOCs. These strains included the Delta strain from India [91,102,117], the Gamma strain reported from Japan that emerged from Brazil [39], and the Beta, the Omicron and its descendant strains, that were detected and reported from southern African research institutes [91,102,103,117]. Therefore, an effective and collaborative BRICS genome surveillance network remains paramount in detecting and reporting new emerging variants and future emerging pathogens in countries that have in the past shown to be the origins where viral variants commonly emerge. This information, in turn, enabled the preparation of healthcare infrastructure and implementation of global public health policies.

China and Russia’s non-pharmaceutical and pharmaceutical intervention initiatives were globally significant. Russia was the first to launch mass vaccination campaigns and produced vaccines used in global immunization campaigns [53]. China’s pandemic intervention was informed by knowledge gained from previous epidemics, which included earlier Coronavirus and major Avian influenza outbreaks [13,14]. This knowledge directed the unprecedented disease intervention, which was later emulated globally, such as setting up fever clinics, limiting public events, and implementing lockdowns. Due to the rigourous containment strategy implemented by China, the epidemiological curve showed a single initial outbreak, followed by smaller localized outbreaks during the first two years of the pandemic. In comparison, more outbreaks and a fully-fledged COVID-19 infectious wave were reported in 2022. The first outbreak of COVID-19 in China was of global importance as it was the first reported outbreak of COVID-19. However, concerns about under-reporting and data transparency remain [79,80].

Genomic data generated by BRICS further drives downstream analyses of global importance. Genome surveillance strategies allow for the identification and monitoring of both VOIs and VOCs. It also supports comparative bioinformatic analyses to identify novel mutations that may lead to potential changes in viral fitness, altered viral behavior, reduced treatment, and vaccine efficiency [118–120]. The cumulation of novel mutations drove increased genomic variability, thus resulting in reduced primer efficiency and reduced quality and coverage of the resulting genomic data [111]. Wide-scale testing, genome surveillance, and bioinformatic analyses drove real-time data generation, which remains important for the timely implementation of informed public health control measures.

BRICS-directed genome surveillance efforts are limited by barriers such as data availability and transparency [15] and ongoing socio-economic and environmental challenges [20]. Addressing data availability and transparency concerns requires the participation of scientific and governmental institutions to accurately disclose case numbers and deaths and submit genomic data to curated and updated databases to achieve effective genome surveillance [79]. Indeed, data disparity between data sources, such as Worldometer, Our World In Data (OWID), and government-specific repositories, hampered effective comparative analysis, limiting the accuracy and informativeness of such investigations. This barrier is exacerbated by the under-reporting of COVID-19 cases and deaths, which remains a challenge not only for BRICS countries but also globally [26]. Moreover, China contributed substantially less genomic data to public repositories such as GISAID **(Tables 1** and **2).** This was thought to be due to political barriers to sharing data related to the outbreak, especially during the early stages of the pandemic when there was still much uncertainty about the virus and its transmission. Civil unrest and armed conflict in BRICS nations may cause reduced testing and limit governmental resources and support to facilitate pandemic intervention and genome surveillance [49]. Moreover, the limited availability of reagents, especially during travel and transport bans, hampers genome surveillance efforts [25]. The consequences of global warming leave BRICS countries vulnerable to large-scale displacement of residents due to flooding, drought, water scarcity, and wildfires [21]. Such environmental challenges compete for public health resource allocation [21]. Concerns regarding climate change-aggravated infectious diseases remain risky for the highly populous BRICS nations. Therefore, effective genome surveillance allowing for timely interventions remains the cornerstone of effective disease intervention to overcome these challenges.

Other pre-existing socioeconomic concerns further undermine effective BRICS pandemic management. These concerns include high levels of migration between countries with ineffective border controls, ongoing viral pandemics [8,10–12], and the consequences of widespread poverty [3,4]. Indeed, in South Africa and India, a high prevalence of the population lives in conditions deemed as extreme poverty (21.2% and 18.9% for India and South Africa, respectively [1]). Additionally, South African residents have a low proportion of fully vaccinated individuals [1,121]. In Russia, previous healthcare challenges include a large proportion of the population older than 65 that is more vulnerable to severe COVID-19 infection. In India, a high prevalence of diabetes accounts for many of the comorbidity concerns [1].

Despite these challenges, intra-BRICS cooperation has proved invaluable throughout the COVID-19 pandemic. This cooperation includes data and scientific transparency, the sharing of resources, and bioinformatic- and laboratory expertise. The NGS-BRICS serves as a platform for an in-depth discussion on the breakthroughs in the laboratory and bioinformatic procedures. Moreover, pathogen surveillance relating to public health threats beyond SARS-CoV-2 includes Monkeypox, Dengue fever, and other arboviral outbreaks. Much of the information gained by the NGS-BRICS is further shared through the NGS-BRICS hosted workshops, which included participants from other African Union member states. These virtual training sessions provide access to detailed knowledge on genome sequencing, phylogenetics and phylodynamics, data visualization, and data repositories that continue to support and facilitate pathogen surveillance for BRICS and other low- and middle-income countries [122]. The ongoing commitment to sharing vaccine resources and scientific and manufacturing expertise was highlighted at the XIII BRICS summit hosted in September 2021 [123]. Here, the signing of the New Delhi declaration recommitted BRICS to combine their resources and ensure appropriate COVID-19 pandemic interventions [124]. During this summit, special emphasis was placed on enhanced vaccine diplomacy and reducing dependency on more economically advanced associations for vaccine production and access. In the interest of an independent health pandemic intervention, the BRICS Vaccine Research and Development platform, hosted by South Africa, will advance the BRICS pandemic intervention [125].

The COVID-19 pandemic revealed important insights and lessons for BRICS. Effective public health interventions for potential future pandemics and disease outbreaks necessitate the development of comprehensive policies and frameworks that address multiple interconnected factors simultaneously. Insights gained from the COVID-19 pandemic included the value of genomic surveillance to direct data-driven decision-making, data transparency, flexible healthcare systems, science-based policy implementations, the dire need to address pre-existing socioeconomic challenges, and collaborative efforts for these countries with their shared economic and healthcare interests. However, a framework is needed to synthesize these lessons and guide their implementation. This framework should incorporate policy recommendations that address the convergence of these insights. It would involve creating policies integrating new knowledge, strengthening cooperation, and addressing healthcare disparities. By developing this framework, BRICS can transform their experiences into strategies, enhancing preparedness for future health threats.

This study provided an overview of the COVID-19 pandemic in the BRICS countries and highlighted key challenges and opportunities for mitigating the impact of COVID-19 and future studies. Based on the findings of this study, future studies can seek to perform comparative analyses on the impact and effectiveness of pharmaceutical and non-pharmaceutical intervention measures on the pandemic, the use of BRICS-generated genomic data to identify potential sources of transmission to and from BRICS countries, to determine whether enhanced surveillance capacity remains effective for the monitor the emergence of new variants as the pandemic continues to progress, a more in-depth evaluation of BRICS health system capacity and pandemic preparedness to address future pandemics, understanding the social determinants of health in different contexts, and exploring how they interact with the pandemic to shape health outcomes. Such studies could inform policies and interventions addressing the root causes of health inequities. Moreover, care should be taken when interpreting the results of this study. The conclusions drawn largely depended on the information Our World In Data provided. OWID’s data sources include official government sources, which may have varying data quality and accuracy levels. This can affect the reliability of the data applied to this study. Although OWID aims to standardize its data collection and reporting, differences in how data is collected and reported across countries remain a concern [126,127]. Moreover, due to limited levels of testing, the total cases reported and the actual mortality due to COVID-19 may remain vastly underestimated; for these reasons, comparative data on seroprevalence will greatly aid the understanding of the accuracy of reported cases per country. Lastly, several parameters are important to consider for a deeper understanding of how the epidemiological contexts compare between these five nations, such as case-to-infection ratio, long COVID rates and mortality rates amongst unvaccinated individuals. We recommend further studies be directed at collecting relevant data and addressing these key epidemiological questions.

The COVID-19 pandemic revealed important insights and lessons for BRICS. Effective public health responses to potential future pandemics and disease outbreaks necessitate the development of comprehensive policies and frameworks that address multiple interconnected factors simultaneously. Insights gained from the COVID-19 pandemic included the value of genomic surveillance to direct data-driven decision-making, data transparency, flexible healthcare systems, science-based policy implementations, the dire need to address pre-existing socioeconomic challenges, and collaborative efforts for these countries with their shared economic and healthcare interests. However, a framework is needed to synthesize these lessons and guide their implementation. This framework should incorporate policy recommendations that address the convergence of these insights. It would involve creating policies integrating new knowledge, strengthening cooperation, and addressing healthcare disparities. By developing this framework, BRICS can transform their experiences into strategies, enhancing preparedness for future health threats.

## Conclusions

BRICS countries and the NGS-BRICS contributed seminal scientific findings during the progression of the COVID-19 pandemic. These contributions included the first whole-genome sequence of SARS-CoV-2, made available at the onset of the pandemic, and the identification and characterization of four of the five VOCs. The BRICS COVID-19 pandemic milestones in 2020 included the first recorded COVID-19 cases, deaths, and high levels of community transmission. Collectively, BRICS nations recorded >105.5 M cases and >1.7 M deaths. Initial BRICS genome surveillance efforts were modest; however, BRICS-generated data increased substantially as the pandemic progressed due to investment in genome surveillance infrastructure. The increased capacity further facilitated reduced turn-around times, allowing real-time data generation to characterize and detect newly emerging variants during 2021 and 2022. Genome surveillance enabled informed pandemic interventions implemented by the BRICS governments and regulatory authorities. Pandemic intervention strategies first implemented by BRICS countries included non-pharmaceutical interventions during the onset of the pandemic that were later applied globally. The enhanced infrastructure and bioinformatic capacity will further support the detection of emerging SARS-CoV-2 variants. They will facilitate pathogen surveillance of pre-existing and ongoing public health threats of global importance.

## Supporting information

S1 Table List of economics/country and region classifications based on the World Health Organization classification criteria. (XLSX)
